# Transmission event of SARS-CoV-2 delta variant reveals multiple vaccine breakthrough infections

**DOI:** 10.1186/s12916-021-02103-4

**Published:** 2021-10-01

**Authors:** Timothy Farinholt, Harsha Doddapaneni, Xiang Qin, Vipin Menon, Qingchang Meng, Ginger Metcalf, Hsu Chao, Marie-Claude Gingras, Vasanthi Avadhanula, Paige Farinholt, Charu Agrawal, Donna M. Muzny, Pedro A. Piedra, Richard A. Gibbs, Joseph Petrosino

**Affiliations:** 1 Alkek Center for Metagenomics and Microbiome Research, Department of Molecular Virology and Microbiology, Baylor College of Medicine, Houston, TX USA; 2 Department of Molecular and Human Genetics, Baylor College of Medicine, Houston, TX USA; 3 Human Genome Sequencing Center, Baylor College of Medicine, Houston, TX USA; 4 Department of Medicine, Baylor College of Medicine, Houston, TX USA

**Keywords:** SARS-CoV-2, Delta variant, B.1.617.2, COVID-19, Infectious disease

## Abstract

**Background:**

This study aims to identify the causative strain of SARS-CoV-2 in a cluster of vaccine breakthroughs. Vaccine breakthrough by a highly transmissible SARS-CoV-2 strain is a risk to global public health.

**Methods:**

Nasopharyngeal swabs from suspected vaccine breakthrough cases were tested for SARS-CoV-2 (severe acute respiratory syndrome coronavirus 2) by qPCR (quantitative polymerase chain reaction) for Wuhan-Hu1 and alpha variant. Positive samples were then sequenced by Swift Normalase Amplicon Panels to determine the causal variant. GATK (genome analysis toolkit) variants were filtered with allele fraction ≥80 and min read depth 30x.

**Results:**

Viral sequencing revealed an infection cluster of 6 vaccinated patients infected with the delta (B.1.617.2) SARS-CoV-2 variant. With no history of vaccine breakthrough, this suggests the delta variant may possess immune evasion in patients that received the Pfizer BNT162b2, Moderna mRNA-1273, and Covaxin BBV152.

**Conclusions:**

Delta variant may pose the highest risk out of any currently circulating SARS-CoV-2 variants, with previously described increased transmissibility over alpha variant and now, possible vaccine breakthrough.

**Funding:**

Parts of this work was supported by the National Institute of Allergy and Infectious Diseases (1U19AI144297) and Baylor College of Medicine internal funding.

**Supplementary Information:**

The online version contains supplementary material available at 10.1186/s12916-021-02103-4.

## Background

High numbers of global SARS-CoV-2 infections have led to the emergence of variants, notably alpha variant (B.1.1.7 UK), beta (B.1.351 S. Africa), gamma (P.1 Brazil), epsilon (B.1.429 California), iota (B.1.526 New York), and now, delta and kappa (B.1.617.2 and B.1.617.1 India). Each of these strains gained advantageous mutations to become a dominant strain, e.g., iota first discovered November 23, 2020, represented 45% of new cases as of February 7, 2021 [1]. Increased transmissibility results from genomic changes such as nonsynonymous mutations in the receptor-binding domain (RBD) of the S-gene (encodes the spike protein) conferring higher binding affinity to host angiotensin-converting enzyme 2 (ACE2) receptors or more efficient cleavage by transmembrane serine protease 2 (TMPRSS2) and subsequently, viral entry [2, 3].

**Fig. 1:**
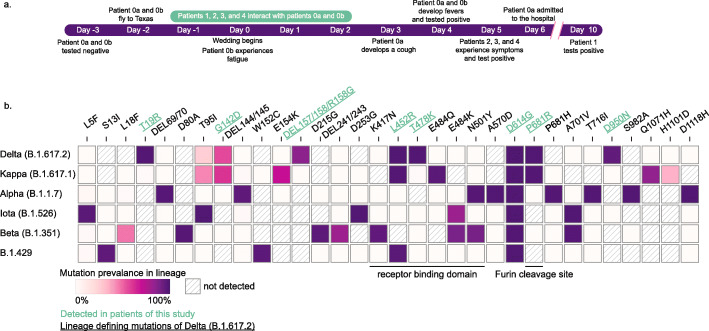
Timeline of events and spike protein mutation prevalence in SARS-CoV-2 variant lineages. **a** Timeline of events denoting positive tests and symptom onset for each patient (if known). **b** Only mutations found in greater than 50% in at least one lineage are displayed. Green text denotes a mutation found in all patients in this report and underlined mutations denote delta variant lineage defining mutations. Figure modified from Outbreak.info [7]

Mutations could also lead to vaccine breakthroughs [4]. The spike protein’s RBD is immunodominant [5], targeted by convalescent sera and vaccine-elicited antibodies (Pfizer BNT162b2 [6]), though evidence suggests a substantial role of the amino-terminal domain (NTD). Mutations in the RBD therefore pose a risk of allowing immune evasion to one or more of the current vaccines [4]. The kappa (B.1.617.2) and delta (B.1.617.2) variants emerged from the Indian state of Maharashtra in December 2020, contributing to a resurgence of cases in the country, representing 70% of daily new cases on May 2, 2021 [7]. The delta variant (B.1.617.2) is now widely circulating in almost 200 countries based on viral sequence data and is classified as a variant of concern by the CDC (centers for disease control and prevention) [7]. The kappa (B.1.617.1) and delta (B.1.617.2) variant lineages are defined by 7 and 8 nonsynonymous mutations in the S protein, respectively (Fig. 1b).

Emergent data suggests partial immunity to the kappa variant (B.1.617.1), as convalescent sera and vaccine-elicited (Pfizer BNT162b2 and Moderna mRNA-1273) antibodies show a 2.3- and 4-fold reduction in neutralization in vitro respectively (noting that this study used protein-pseudotyped lentiviruses lacking the T478K mutation found in delta variant [B.1.617.2]) [8]. A test-negative case-control study estimated the effectiveness of vaccination (2 weeks post-second vaccination) against symptomatic disease by delta variant (B.1.617.2) to be as high as 88% for Pfizer BNT162b2 in the UK (compared to 93% for alpha variant [B.1.1.7]) [9].

Here, we describe the transmission of the delta variant (B.1.617.2) of SARS-CoV-2, between family members associated with events surrounding a wedding with <100 attendees, near Houston, Texas. Attendance to formal wedding events required guests to be fully vaccinated and took place outdoors in a large, open-air tent. To date, 6 individuals have tested positive for SARS-CoV-2, all patients were symptomatic, one patient severely enough to receive monoclonal antibody infusion treatment (Regeneron Pharmaceuticals Inc.), and one patient has died. Encounter timings and viral sequence similarities suggest the strain containing the delta variant (B.1.617.2) was transmitted to wedding guests from two patients traveling from abroad. With no history of vaccine failure in these patients, our observations suggest these are true cases of vaccine breakthrough, mediated by the delta variant (B.1.617.2).

## Methods

### Specimen collection and ethical considerations

All individuals were initially tested by third-party SARS-CoV-2 testing sites. Verbal consent was obtained, and nasopharyngeal swabs were collected by a physician or nurse as close to the first positive test as possible. Samples were submitted to the Alkek Center for Metagenomics and Microbiome Research for RNA extraction and the Human Genome Sequencing Center for qPCR confirmation. Protocols for collection, qPCR testing, and whole-genome sequencing were approved by the Baylor College of Medicine Institutional Review Board (H-47423).

### cDNA synthesis and amplicon libraries

RNA extracted from nasopharyngeal swabs of six individuals that tested positive for SARS-COV-2 using qPCR was converted to the 1st-strand cDNA using SuperScript™ IV First-Strand Synthesis System (Thermo Fisher, Cat. No. 18091050). The 1st-strand cDNA reaction was performed starting with 10 μl of the total RNA in a 25-μl reaction mix, which was incubated at 23°C for 10 min followed by 50°C for 50 min. The resulting 1st-strand cDNA was then diluted with DEPC-treated water, where for two samples with Ct<24 cDNA was diluted 20 times and for the remaining four samples, with Ct>24, cDNA was diluted 2 times, respectively. This diluted 1st-strand cDNA (10 ul) was used as input for amplification of the SARS-CoV-2 viral genome, using the SARS-CoV-2 Additional Genome Coverage Panel (Cat#COVG1V2-96). This panel was designed against the SARS-CoV-2 Wuhan-Hu-1 strain (NC_045512.2) and has 345 amplicons of 116-255 bp (average 150 bp) that cover 99.7% (29,828 of 29,903 total bases) of the genome.

These amplicons come in a single tube, and the workflow involves two rounds of PCR, a multiplex PCR (4 + 18 cycles) and the indexing PCR (9 cycles) to generate sequence-ready libraries. The reaction mixes and the thermocycler conditions were performed according to the Swift Normalase® Amplicon Panels (SNAP) Workflow. Libraries were barcoded with 8bp unique dual indices at the Indexing PCR. For library normalization, the 2-nM Normalase I protocol was performed on libraries individually, followed by pooling 5 μl of each post-Normalase I library to perform Normalase II reaction, which results in sequence ready library pool. Before sequencing, the normalized library pool concentration was measured using qPCR with KAPA Library Quantification Kits (Roche, KK4835, 07960204001).

### Illumina sequencing

The pooled SARS-CoV-2 amplicon libraries were sequenced on Illumina NovaSeq 6000 S4 flowcell to generate 2x150bp reads.

### Swift amplicon data analysis

Swift amplicon data were analyzed using Swift Biosciences Sarscov2 analysis pipeline (https://github.com/swiftbiosciences/sarscov2analysis_docker) with a minimum read coverage depth of 3. The GATK variants were next filtered with allele fraction ≥80 and min read depth 30x [10, 11]. Swift analysis pipeline produced variant vcf file, consensus genome, pangolin lineage, and Nextclade assignment (https://clades.nextstrain.org/). Variant vcf from Swift amplicon data was also annotated using SnpEff [11] [12].

### Phylogenetic analysis

Sequences for the designated variants of concern and variants of interest by the centers for disease control (CDC) were downloaded from GISAID (global initiative on sharing all influenza data) on June 2, 2021 [13]. All samples downloaded from GISAID were analyzed using Pangolin V3.0.3 with pangoLEARN 2021-05-27 [14] to ensure that the variant designation assigned by GISAID is accurate. Global alignment of 334 sequences including the sequences from the current study was done using MAFFT v7.480 [15]. Maximum-likelihood phylogenetic tree with bootstrap (5000) was generated using IQ-Tree V2.1.2 [16, 17]. Annotation and visualization of the tree were carried out by using FigTree v1.4.4 (http://tree.bio.ed.ac.uk/software/figtree/). Clades were labeled with the WHO nomenclature.

## Results

In early April 2021 (day 2, Fig. 1a), patient 0a, a man with no comorbidities, and a woman, patient 0b, traveled to attend a wedding outside of Houston, Texas (designated 0a and 0b due to difficulty establishing true patient 0). Both tested negative for SARS-CoV-2 by qPCR as part of the pre-flight criteria (day 3). Formal wedding events were held outdoors and in a large open-air tent. Attendance required full vaccination, but masks were optional (patients 0a and 0b traveled to Houston 7 days after their second doses of Covaxin BBV152, Table 1). Patients 1–4 confirmed having close encounters with patients 0a and 0b at the wedding. Events were attended by a range of guests, indoor event of ~25 people on day 1, outdoor event <100 on day 0, outdoor event <100 on day 1, and an indoor event of ~25 on day 2 (Fig. 1a). All patients interacted for several hours during each event. Patients 1 and 2 traveled to events in the same vehicle.

**Table 1:** Patient demographics, vaccine history, and symptoms

Sample	Sex	Age	Height (cm)	Weight (kg)	Vaccine received	First dose	Second dose	Symptoms	Comorbidities†	N1 qPCR Ct	Day of positive test	Clinical outcome
Patient 0a	Male	67–69	177.8	81.6	Covaxin BBV152	2021-03-02	2021-03-31	Fever, cough, body aches, fatigue, loss of taste and/or smell, Shortness of breath at rest, Shortness of breath with activity	None	29	Day 4	Deceased
Patient 0b	Female	65–70	152.4	53	Covaxin BBV152	2021-03-02	2021-03-30	Fever, cough, body aches, fatigue, loss of taste and/or smell, Shortness of breath at rest, Shortness of breath with activity	Diabetes	27	Day 4	Recovered
Patient 1	Male	60–66	172	68	Pfizer BNT162b2	2021-12-30	2021-01-20	Cough, fatigue	None	17	Day 4	Recovered
Patient 2	Male	59–64	170.1	77	Pfizer BNT162b2	2021-01-07	2021-01-28	Fever, cough, fatigue	Hypertension	24	Day 5	Recovered
Patient 3	Female	51–56	155	63.5	Moderna mRNA-1273	2020-12-30	2021-01-26	Fever, cough, body aches, fatigue, loss of taste and/or smell	Overweight	25	Day 5	Recovered
Patient 4	Female	51–56	162.6	88.5	Moderna mRNA-1273	2021-01-04	2021-02-01	Fatigue, loss of taste and/or smell	Overweight	22	Day 5	Recovered

^†^As defined by the Centers for Disease Control and Prevention

On the evening of day 0, patient 0b complained of fatigue but associated it with diabetes and jet lag. Patient 0a developed a cough on day 2 and both him and patient 0b developed a fever on day 4. Patients 0a and 0b tested positive for SARS-CoV-2 by nasal swab qPCR on day 4 at a third-party site. Patient 0a’s symptoms progressed over the following days and were admitted to the hospital on day 6. He was transferred to an intensive care unit in the Texas Medical Center with worsening symptoms. Thirty-six days after the wedding, patient 0a died from complications of COVID-19 (coronavirus disease of 2019).

Following the wedding, four additional guests tested positive for SARS-CoV-2 after confirmed interactions with patients 0a and 0b. All positive patients received Pfizer BNT162b2, Moderna mRNA-1273, or Covaxin BBV152 (Table 1). Patients 2, 3, and 4 developed COVID-1 symptoms and tested positive on day 5 (Fig. 1a and Table 1). Patient 1, who received the Pfizer BNT162b2 vaccine developed severe symptoms and was admitted to the hospital for monoclonal antibody infusion treatment (Regeneron Pharmaceuticals Inc.). The number of vaccine breakthroughs resulting in COVID-19 symptoms suggested the patients were carrying a SARS-CoV-2 variant.

To characterize the variant, the total RNA was extracted from nasopharyngeal swabs of each of the 6 patients. All positive for SARS-CoV-2 Wuhan-Hu-1 and negative for alpha variant by qPCR (Table 1). Human RNase P (RP) gene control values suggested sampling of patients and RNA isolation were performed optimally. Amplicon libraries were successfully prepared from all 6 qPCR-positive samples (N1 Ct value 17–29, Table 1), with 900,754–2,381,756 pass-filter reads generated using Swift Biosciences Sarscov2 analysis pipeline (Supplementary Table 1). Median sequence coverage ranged from 2085x to 12,932x with >99.7% of the genome covered at 40x or greater.

**Fig. 2:**
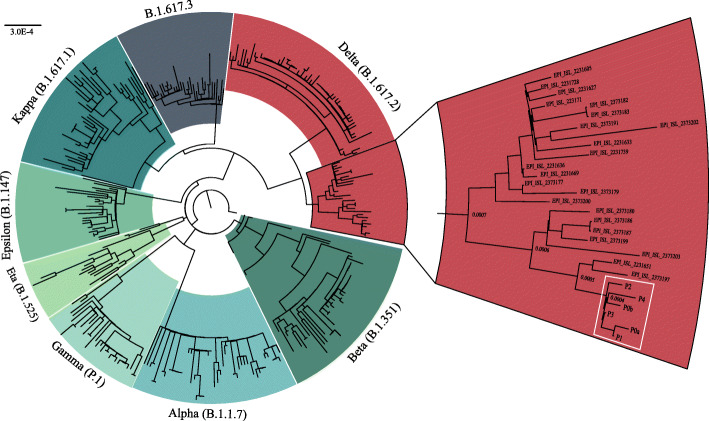
Phylogenetic analysis of SARS-CoV-2 variants. All patients (white box) cluster in a subclade of the delta variant (red). Sequences obtained from GISAID

All 6 samples were identified as the SARS-CoV-2 delta variant (B.1.617.2) based on the presence of the 10 mutations listed by the CDC’s “Selected Characteristics of SARS-CoV-2 Variants of Interest.” These mutations, located on the S protein, were T19R (G142D), 156del, 157del, R158G, L452R, T478K, D614G, P681R, and D950N. 156del, 157del, and R158G are annotated as a single mutation (S:GAGTTCA22028G:Glu156_Arg158delinsGly) due to their proximity (Fig. 1b). Identified mutations align with the most prevalent mutations in other delta variant sequences from GISAID (Fig. 1b green underlined text). Phylogenetic analysis places each patient sample in a subclade of the delta variant (white box, Fig. 2).

## Discussion

Ending the current SARS-CoV-2 pandemic requires limiting the spread through continued vigilance of masking, social distancing, and vaccination. Variants emerge from areas experiencing uncontrolled viral spread and display increased transmissibility due to mutations in the spike protein. These mutations may occur in the antigenic region of the RBD, altering binding sites for vaccine-elicited antibodies. Mutations such as the ones found in the Kappa variant provide partial resistance to antibody neutralization (Pfizer BNT162b2, Moderna mRNA-1273, Regeneron [8], and Covaxin BBV152 [18]), likely due to changes in epitope sequence.

Vaccine breakthrough by highly transmissible variants (delta variant up to 60% more transmissible than alpha variant [19]) could lead to significant setbacks in pandemic control efforts, requiring renewed social distancing and masking efforts. Significant vaccine breakthroughs could necessitate vaccine boosters or targeted lockdowns to reduce the spread of infection. An analysis of spike protein epitopes found several antigenic regions (IDa-IDi, Zhange et al.). Delta variant (B.1.617.2) spike protein contains mutations in three of these regions (450–469 IDf, 480–499 IDg, and 522–646 IDh, Fig. 1) possibly resulting in decreased neutralization by vaccine-elicited antibodies.

According to the cases presented in this study, antibodies elicited in patients receiving Pfizer BNT162b2, Moderna mRNA-1273, and Covaxin BBV152 may provide decreased immunity to the delta variant (B.1.617.2). Being among the first to identify the delta variant (B.1.617.2) in the USA (first identified March 2021 and accounting for >60% of all new cases as of July 15, 2021 [7]), we anticipate additional cases will be identified and provide a more accurate measure of vaccine breakthrough. It is possible that some individuals in this study failed to produce an effective immune response to their immunization. Without further serological studies, we are unable to conclude that each patient’s vaccination yielded an appropriate response. However, none of the patients had previously contracted targets of previous vaccinations, suggesting a functioning immune response. Further study is needed to confirm appropriate antibody titers are measured in these patients.

Recent data suggests two doses of Pfizer BNT162b2 and Moderna mRNA-1273 provide 88% protection from severe COVID-19 and 96% protection from hospitalization. The largest effect the mutations contained in the delta variant (B.1.617.2) genome seem to have is the protection provided by a single dose of these vaccines, estimated to be 33% effective against the delta variant (B.1.617.2) compared to 51% against the alpha variant (B.1.1.7) [9]. Being only 7–8 days from their second dose at the likely point of transmission, patient 0a’s antibody titers may not have been sufficient to prevent severe COVID-19.

The cases presented in this study agree with recent studies, and incubation time (time from exposure to symptom onset) for the delta variant (B.1.617.2) remains similar to previous variants (4–6 days).

## Conclusions

Our observations support continued efforts to generate SARS-CoV-2 genomic sequences from positive patient samples, to identify possible vaccine breakthrough mutations. The continued effectiveness of vaccine-elicited antibodies towards SARS-CoV-2 variants highlights the importance of vaccination efforts. Mathematical modeling of pandemic cessation suggests at 75% population coverage of a vaccine with 80% efficacy is sufficient for a virus with a reproduction number (*R*_0_) of 3.5 [20]. The delta variant (B.1.617.2) with an *R*_0_ of 4–8 would require 90% vaccination coverage with a vaccination efficacy of 90% [21]. Slowing the spread could prevent the emergence of future variants, hastening the end of this pandemic.

## Supplementary Information

**Additional file 1:.** Supplementary Figure 1. Mutation metrics from sequencing results for all 6 samples. Figure generated using Coronapp [12]

**Additional file 2:.** Supplementary table 1. Swift Ampilicon Normalize Panel sequencing metrics

## Data Availability

The datasets generated and analyzed during the current study are available from the corresponding author on reasonable request. Sequence data available on GISAID under IDs: hCoV-19/USA/TX_GCID_1004144931/2021; EPI_ISL_2529850hCoV-19/USA/TX_GCID_1004144931/2021; EPI_ISL_2529850hCoV-19/USA/TX_GCID_1004155249/2021; EPI_ISL_2529852hCoV-19/USA/TX_GCID_1004155261/2021; EPI_ISL_2529854hCoV-19/USA/TX_GCID_1004155268/2021; EPI_ISL_2529856hCoV-19/USA/TX_GCID_1004155259/2021; EPI_ISL_2529858hCoV-19/USA/TX_GCID_1004155265/2021; EPI_ISL_2529861
