# Supplementary figures and images for: Transmission event of SARS-CoV-2 delta variant reveals multiple vaccine breakthrough infections
Source: BMC Med. 2021 Oct 1;19:255. doi: 10.1186/s12916-021-02103-4 (PMC8483940; doi:10.1186/s12916-021-02103-4)

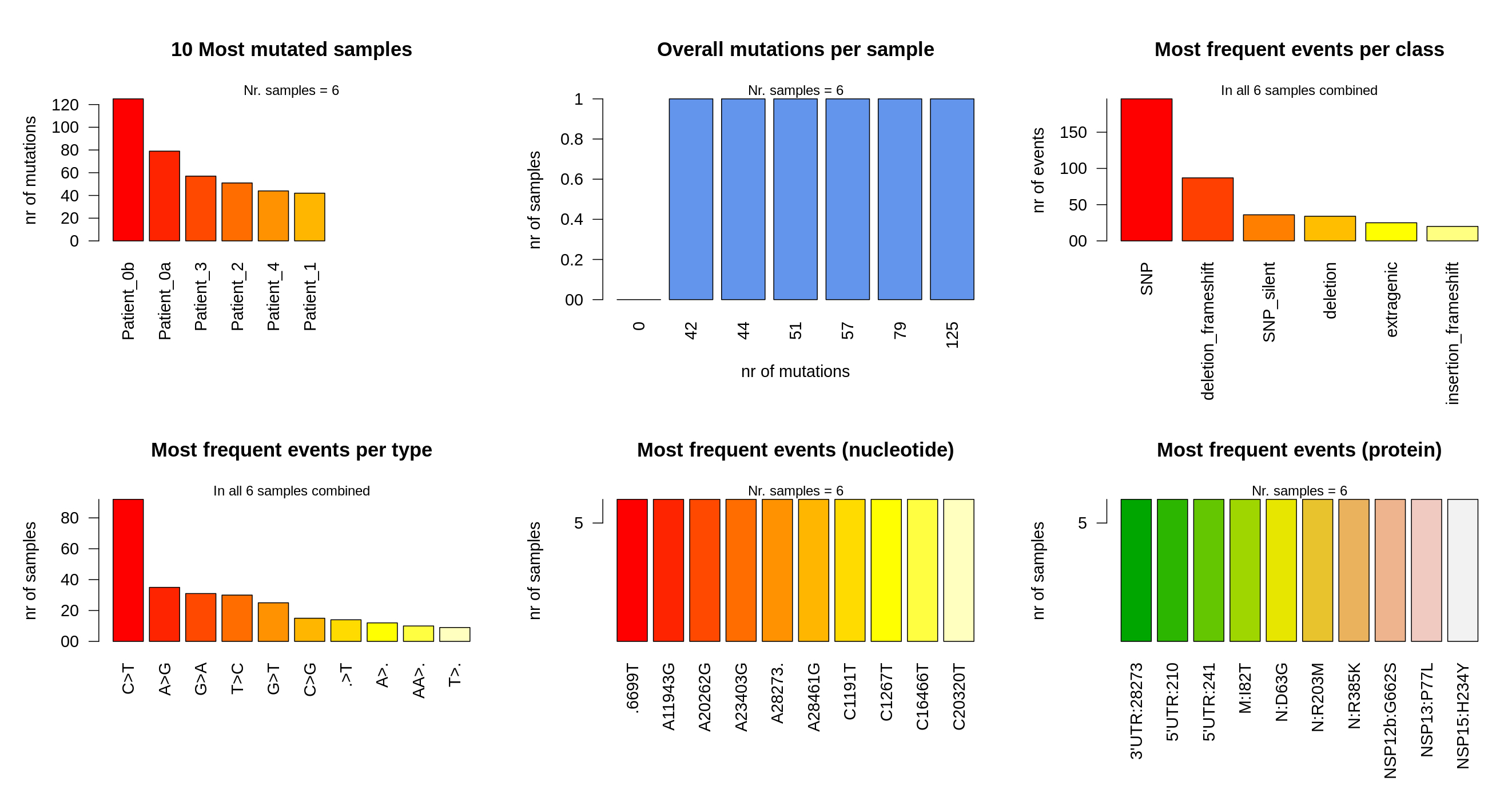

Supplement: Supplementary file 1 — Additional file 1:. Supplementary Figure 1. Mutation metrics from sequencing results for all 6 samples. Figure generated using Coronapp [12] [file 12916_2021_2103_MOESM1_ESM.jpg]
