# Supplementary material for: Transmission event of SARS-CoV-2 delta variant reveals multiple vaccine breakthrough infections
Source: BMC Med. 2021 Oct 1;19:255. doi: 10.1186/s12916-021-02103-4 (PMC8483940; doi:10.1186/s12916-021-02103-4)
Supplement: Supplementary file 2 — Additional file 2:. Supplementary table 1. Swift Ampilicon Normalize Panel sequencing metrics [file 12916_2021_2103_MOESM2_ESM.pdf]

Supplementary table 1. Swift Amplicon Normalase Panel sequencing metrics

| Sample | Reads passed filter | Yield (Mb) | Mean | Stdev | Median | Maximum | Bases without coverage | 0x % | >=5x % | >=10x % | >=20x % | >=40x % |
|---|---|---|---|---|---|---|---|---|---|---|---|---|
| Patient 0a | 900,754 | 272 | 2941.96 | 3405.34 | 2085 | 216863 | 46 | 0.15 | 99.85 | 99.84 | 99.83 | 99.8 |
| Patient 0b | 2,381,756 | 719 | 15470.4 | 11416.18 | 12932 | 108010 | 43 | 0.14 | 99.85 | 99.85 | 99.84 | 99.84 |
| Patient 3 | 1,458,602 | 440 | 9711.2 | 8845.54 | 7534 | 81585 | 42 | 0.14 | 99.83 | 99.81 | 99.8 | 99.72 |
| Patient 1 | 1,424,114 | 430 | 8651.92 | 14635.48 | 4457 | 159997 | 40 | 0.13 | 99.84 | 99.84 | 99.83 | 99.72 |
| Patient 4 | 1,501,130 | 453 | 9067.53 | 8965.74 | 6690 | 119267 | 42 | 0.14 | 99.86 | 99.84 | 99.83 | 99.8 |
| Patient 2 | 1,710,539 | 517 | 11420.22 | 10814.68 | 8203 | 91027 | 41 | 0.14 | 99.85 | 99.84 | 99.83 | 99.72 |
